# Some Dangers of Spring

**Published:** 1887-05-07

**Authors:** 


### SOME DANGERS OF SPRING.

BY A FAMILY DOCTOR.

"CAST na a cloot, till May be oot," says the Scotch proverb. What is a proverb? Is it merely the happy inspiration of a moment? As a rule, No. It is a slowly gathered result of experience; and experience is of all things one of the most valuable. Many people think Solomon must have been a very wise man, because he wrote so many wise proverbs. He probably did not write very many at all. We read that "he sought out and set in order many proverbs." He showed as much strong sense and wisdom by so doing, though perhaps not so much originality, as if he had actually struck the proverbs newly out of the mint of his own genius.

"Cast na a cloot, till May be oot." It is one of the most frequently used of the Scotch proverbs; and the Scotch are a shrewd people. That means that the proverb is worth thinking about. Don't change your winter clothing until the end of May. It sounds a decidedly uncomfortable commandment. Some May, and even April days are scorchingly hot; and must we wear our thick overcoats, flannel jerseys, lambswool socks, and other winter paraphernalia when the thermometer stands at 90 deg., simply because some pragmatical old Scotchman once thought it wise to put a little of his experience into the form of a common people's proverb? No, sir! Certainly not. A proverb is a very good thing, but not a thing for men and women to make themselves slaves to. Very few sayings indeed are of such a character as to demand at once implicit and unquestioning obedience without reflection. Even St. Paul rarely asked that for his utterances. "Consider what I say," said he, "and the Lord give thee understanding." And that is the use which should be made of proverbs: they should be considered.

Why should the accumulated experience of the Scottish people formulate such a proverb as the one at the commencement of this paper? Because from generation to generation, people who rashly and hastily changed their winter clothing in April and May, paid dearly for it. Some of them got bronchitis, some pleurisy, some inflammation of the lungs, some rheumatic fever, some disordered livers, and some neuralgias in various parts of the body. No doubt these sufferers, when they got better, often compared notes, and said to each other, what a pity it was they had not rather endured the increasing heats of the spring, or at any rate changed their winter clothing more slowly and cautiously, than suffered such severe and often dangerous illnesses. These discussions were probably repeated time after time, until at length some village patriarch more given to oracular utterances than his neighbours, gathered up all their various wisdom and experience into one brief and pithy sentence; a sentence which was found by all to be so apt an expression of their own views of the matter, that it then and there crystallised into the now familiar and universally accepted proverb, "Cast na a cloot, till May be oot."

One of the principal dangers of spring is the too early or the ill-considered change of clothing. The great point to be borne in mind is, that the change should be gradual. In this country the wealthier classes alone have garments suited in texture, colour, and cut for every season. The lower middle, and poorer people have but two sets, the one for summer and the other for winter. Well, but a little trouble can soon arrange that these also shall be able to play at chess with the seasons, and make the proper move at the proper time. A little trouble! How some people hate trouble! They would rather run the risk of pleurisy or rheumatic fever than get up from the chair to shut a door, or spend an hour in repairing a half-worn-out jersey. Pleurisies, rheumatisms, inflammations of the lungs, may be got by a quarter of an hour's carelessness or idleness, but the results may, and not seldom do, remain with the patient for a lifetime. Want of care often does ten times more harm than want of knowledge. To everybody, but especially to the aged, the young and the delicate, we would say, "Prevention is a thousand times better than cure; and during the months of March, April, and May use your senses in finding out the character of the weather and the quarter where the wind sits; use your best judgment as to the kind of clothing you should wear every day; and above all, do not grudge a little trouble in making, mending, or changing clothes when the varying conditions of the climate demand it." We began with a Scotch proverb; we will end with one which is both Scotch, English, and universal—"A stitch in time saves nine."

